# Dynamic imaging for dynamic lung events

**DOI:** 10.1007/s10877-021-00775-0

**Published:** 2021-10-29

**Authors:** Tobias Becher, Dirk Schädler, Inéz Frerichs

**Affiliations:** Klinik für Anästhesiologie und Operative Intensivmedizin, Universitätsklinikum Schleswig-Holstein, Campus Kiel, Kiel, Germany

Since the groundbreaking experimental studies on ventilator-induced lung injury (VILI) performed in the 1990s [1], barotrauma, volutrauma and atelectrauma have been recognized as major causes of morbidity and mortality in mechanically ventilated patients with respiratory failure. In addition to the reduction in mortality provided by ventilation with lower tidal volumes [2], early restoration of spontaneous breathing has been shown to be beneficial in improving oxygen delivery and in shortening the duration of mechanical ventilation in patients with mildly impaired gas exchange [3]. Letting the patient breathe spontaneously through the ventilator may thus contribute to reducing the exposure time during which patients are mechanically ventilated and therefore at risk for developing VILI. The rationale for restoring early spontaneous breathing was further strengthened by demonstrating the long-term benefits of daily awakening and spontaneous breathing trials [4]. On the other hand, clinical research in patients with moderate-to-severe ARDS demonstrated that preventing spontaneous breathing through the use of neuromuscular blocking agents may reduce biotrauma [5] and lead to improved clinical outcomes [6]. These findings were supported by experimental studies demonstrating that high transpulmonary pressure swings associated with strong spontaneous breathing efforts may lead to worsening of lung injury [7] and produce local overstretch in inhomogeneously injured lungs [8]. The potentially injurious effects of vigorous spontaneous breathing in the presence of lung injury have since then been summarized under the still relatively new term “patient self-inflicted lung injury” (P-SILI) [9]. In patients with respiratory failure, the potential benefits of light sedation and spontaneous breathing must thus be balanced against the risks of P-SILI caused by excessive patient efforts, especially in the presence of inhomogeneous lung injury. This trade-off can also explain why a recent randomized controlled trial found no difference in outcome between a strategy of light sedation and spontaneous breathing, as compared to a strategy with deep sedation and neuromuscular blockade in patients with ARDS [10]. The possible benefits of neuromuscular blocking agents in preventing P-SILI may, on average, have been offset by the harm caused by the necessary deeper sedation levels. The clinical challenge is therefore to find a method for identifying individual patients who are at increased risk for developing P-SILI, as in these patients, the potential benefits provided by a strategy of deep sedation and muscle relaxation may outweigh the adverse effects this strategy inevitably entails.

In this issue of the Journal of Clinical Monitoring and Computing, Pulletz and coworkers present an innovative approach for determining the dynamic regional relative strain (DRRS) with electrical impedance tomography (EIT) during unassisted spontaneous breathing [11]. DRRS is obtained by calculating the ratio between normalized pixel values of tidal impedance change and end-expiratory lung impedance (EELI). This method has the potential of identifying regional overdistension during unassisted spontaneous breathing: higher values of DRRS indicate lung areas with a high ratio between tidal volume and end-expiratory lung volume (strain). As high inspiratory strain is a known risk factor for development of volutrauma [12], high values of DRRS could identify lung areas at risk for volutrauma, even during unassisted spontaneous breathing.

In their present study, Pulletz and coworkers compared DRRS between a group of spontaneously breathing patients suffering from COVID-19 and a control group of healthy volunteers. They found that elevated pixel values of DRRS were more frequent in COVID-19 patients as compared to healthy controls. As COVID-19 patients may be at increased risk for developing P-SILI [13], these data indicate that DRRS could prove to be a useful marker for early recognition of potentially injurious spontaneous efforts.

The potential of the new method, however, comes with a few caveats: As a consequence of the normalization procedure, the average DRRS value of the patient’s lungs is always close to 1, regardless of the severity of lung damage or the vigorousness of spontaneous efforts. DRRS therefore only provides regional information on lung areas that are more distended in comparison to the rest of this individual patient’s lungs. If, as a consequence of inhomogeneous lung damage, some lung regions exhibit an above-average stretch, DRRS for these lung regions will assume higher values, indicating relative regional overdistension. If, on the other hand, lung stretch is distributed homogeneously across the whole lung without any particularly overdistended areas, DRRS may remain close to 1 even if the entire lung is already dangerously close to overdistension. Moreover, as the calculation of DRRS is based on a comparison between normalized pixel values of tidal impedance change and EELI, it may be influenced by the application of PEEP. High levels of PEEP may thus lead to potentially lower values of DRRS in lung regions that are already overdistended at end-expiration and whose tidal impedance change is therefore low in comparison to their already-high EELI.

Despite these caveats, Pulletz and coworkers present an intriguing approach that adresses a previously unresolved clinical challenge: identifying patients at risk for P-SILI while they are still breathing spontaneously without assistance.

## Data Availability

Not applicable.
